# Determinants for the implementation of person-centered tools for workers with chronic health conditions: a mixed-method study using the Tailored Implementation for Chronic Diseases checklist

**DOI:** 10.1186/s12889-021-11047-6

**Published:** 2021-06-07

**Authors:** N. Zipfel, B. Horreh, C. T. J. Hulshof, A. Suman, A. G. E. M. de Boer, S. J. van der Burg-Vermeulen

**Affiliations:** 1 Department of Public and Occupational Health, Coronel Institute of Occupational Health, Amsterdam Public Health Research Institute, Amsterdam University Medical Centers, University of Amsterdam, PO Box 22700, 1100 DE Amsterdam, The Netherlands; 2 Julius Center for Health Sciences and Primary Care, University Medical Center Utrecht, Utrecht University, Utrecht, The Netherlands

**Keywords:** Person-centered tools, Occupational health, Chronic health conditions, Implementation research

## Abstract

**Background:**

The aim was to identify the most important determinants of practice for the implementation of person-centered tools which enhance work participation for patients with chronic health conditions.

**Methods:**

A mixed-method study was conducted consisting of semi-structured interviews, a focus group and a survey. Various stakeholders were involved including (representatives of) workers with chronic health conditions, insurance physicians, occupational physicians, other healthcare professionals, researchers, employers, and policymakers. The semi-structured interviews were performed to identify implementation determinants, followed by a focus group to validate resulting determinants. To conclude, a survey was conducted to select the most important implementation determinants through prioritization by ranking the order of importance. The Tailored Implementation of Chronic Diseases checklist (TICD) was used as concept-driven coding frame for the qualitative analysis of the interviews and focus group. The self-developed survey was based on the domains of the TICD. The survey was analyzed by frequency count of first ranking of determinants per and between domains of the TICD.

**Results:**

Various stakeholders participated (*N* = 27) in the interviews and focus group. The qualitative data retrieved yielded a list of determinants with additional in-depth themes according to the TICD. For the selection of the most important determinants, a survey with 101 respondents was conducted, consisting of occupational physicians, insurance physicians and workers with a chronic health condition. From the seven domains of the TICD, respondents emphasized the importance of taking into account the needs and factors associated with workers with a chronic health condition as this determinant ranked highest. Taking into account the individual needs and wishes of workers was mentioned to enable successful implementation, whereas stress of the workers was indicated to impede implementation. Other important determinants included ‘being able to work with the tools’ in terms of time and usability or ‘cognitions, beliefs and attitudes of occupational and insurance physicians’ to be able to use the tools.

**Conclusion:**

This study identified the most important determinants from the perspective of various stakeholders involved in the implementation of client-centered tools in occupational health for workers with chronic health conditions. Furthermore, by prioritizing the most important determinants, targeted implementation strategies can be developed.

**Supplementary Information:**

The online version contains supplementary material available at 10.1186/s12889-021-11047-6.

## Background

The prevalence of chronic health conditions is rising rapidly worldwide among all age groups [1]. With this increase, chronic health conditions are becoming more prevalent within the working population [2]. In the Netherlands, in 2019 approximately 36% of workers experienced a chronic health condition [3]. From these workers around 43% experienced slight impediment at work while 10% reported significant barriers due to the chronic health conditions [3]. In order to enhance work ability and participation when having a chronic health condition, person-centered care (PCC) is becoming an essential part of current healthcare systems [4]. The concept of PCC entails a shift towards the perspective of the person functioning in daily living with a disease or impairment in order to enhance coping and management of the disease [5]. PCC improves communication between care providers and patients on treatment and care decisions [6], and might also be of added value to occupational health physicians supportive relationships with their patients to improve work participation guidance and sustainable work ability. In occupational health, patients are often referred to as clients [7] and client-centered practice (CCP) has been defined as “client-centered practice is a process in which the client is the focal point” [8]. Both concepts share common characteristics as considering the patient’s values, preferences and goals for decision-making [9]. In PCC a patient is considered as someone who needs to be treated due to illness. Whereas in CCP, also often referred to as person-centered care, the entire person including the context and surroundings are considered [9].

To enhance work participation, we developed CCP tools to support occupational physicians and insurance physicians in providing optimal work-related guidance and assessment for workers with chronic health conditions [10–12]. These tools aim at (1) strengthening self-control of workers with chronic health conditions, (2) understanding worker’s person-related factors associated with work participation such as cognitions and perceptions, and (3) involving significant others in supporting work participation. A detailed description of the tools can be found in Table 1. The current study was part of a larger research project involving a consortium of various stakeholders and end-users for the co-creation of the CCP tools. An earlier study stated that two objectives need to be met to implement occupational health interventions; the first being that the intervention should be evidence based and the second that it should fit the needs of the organization and the workers [13]. The CCP tools were tested in experimental study designs to be published in separate publications. Additionally, the CCP tools were developed based on existing knowledge from literature reviews and primary data from OPs and IPs [11, 12, 14–16]. Therefore, making the tools evidence-based in co-creation with the end-users meets the objectives to implement occupational health interventions. Involving end-users in the development and implementation of CCP tools is highly valuable for implementation success and upscaling. An earlier study underlines the importance of the involvement of the end-user, specifically the patient, when developing tools focused on self-efficacy [17]. Involvement of end-users in the form of all involved stakeholders, interested parties and workers can lead to the development of a broad spectrum of CCP tools.

**Table 1:** Description of the CCP tools

**1) Strengthening self-control of workers with chronic health conditions**	Using the Intervention Mapping methodology, a tool was developed for occupational physicians to guide organizations to be supportive for workers with a chronic health condition in increasing their self-control. The tool consists of three parts: a training phase, an application phase and a feedback phase. In the training, occupational physicians were trained as process leaders to help organizations apply the participatory approach at an organizational level. The participatory approach is a stepwise process in which the organization identifies and prioritizes problems and solutions with regards to supporting workers with chronic health conditions in increasing their self-control. A key element of the participatory approach is the involvement of stakeholders throughout the process, such as managers, HR managers and workers with a chronic health condition. The outcome of the participatory approach is an action plan consisting of different interventions that is embedded in organizational policy, and leads to adaptation of the work environment so that problems related to the workplace can be discussed and work-related consequences of chronic health conditions can be prevented as much as possible. In the application phase, occupational physicians applied their new skills in an organization, and in the feedback phase, meetings were organized in which occupational physicians can exchange their experiences with applying their new skills.
**2) Involving person-related factors (cognitions and perceptions) in the occupational health management and work disability assessment**	The goal of this training is to teach occupational health professionals (e.g. occupational and insurance physicians) how to involve the worker’s cognitions and perceptions in occupational health management and work disability assessment of workers with a chronic health condition. During the training participants acquire knowledge of 10 cognitions and perceptions important for work participation, how to obtain information concerning these person-related factors, the course of the conversation on these factors and intervening on limiting cognitions and perceptions when necessary. The participants receive a conversation tool which can help them to apply the acquired skills during the training in practice. The information provided during the training, is based on four previously conducted studies; a systematic review about important cognitions and perceptions for work participation, a survey study among physicians and a focus group study among workers with chronic health problems about how to obtain information concerning cognitions and perceptions and a scoping review on interventions. The training has a duration of 4.5 h and includes classical presentations and different exercises to practice with the acquired skills.
**3) Involving significant others in the work re-integration process of workers with a chronic disease**	Workers with a chronic health condition may be better supported in their recovery and work re-integration when their significant others such as a partner, family members or friends are more involved in the re-integration process. Using an e-learning approach, occupational and insurance physicians learn how they can obtain insight in the influence of significant others and involve them in the re-integration process to better support the worker in recovery and re-integration. The content of the e-learning was in part based on the results of previous studies that sought to gain insight in relevant cognitive-behavioral factors of significant others [16], OHPs’ current practices [10] and stakeholders’ views on involving significant in occupational health care. In addition, content was based on additional research on current practices with regard to involving significant others in related fields and available literature on the topics addressed within the e-learning. The e-learning is accompanied by a conversation tool, consisting of: (1) a document with an overview of the key messages and best-practice recommendations from the e-learning, (2) validated questionnaires and open questions that physicians can use to obtain insight in illness perceptions and coping of workers and their significant others, (3) informational folders to facilitate adaptive illness perceptions, and (4) a flyer to facilitate communication and dyadic coping.

For successful implementation of CCP tools, understanding important determinants for the implementation is crucial [18]. Earlier studies identified several barriers for the implementation of evidence-based interventions in occupational healthcare practice [19–21]. These include barriers concerning the sociopolitical context; the organizational level such as resources, management support, and cultural compatibility; and the occupational physician level including personal factors, support from colleagues, expectations and relative advantages [19, 21]. These studies show the importance of identifying determinants that might affect the implementation besides demonstrating effectiveness [22]. However, none of these studies made use of a systematic framework for mapping possible facilitators and barriers to the implementation. Although previous implementation studies may have been thorough and detailed, chance is that barriers and facilitators have gone unexamined due to a lack of a systematic framework for mapping implementation determinants. In our study, we aim to fill this methodological gap. The TICD checklist offers the possibility and structure for examining implementation determinants in all domains of relevance to improve healthcare for patients with chronic health conditions. The ‘Tailored Implementation for Chronic Diseases’ (TICD) checklist offers a pre-defined framework of determinants of practice to guide successful implementation and understand important factors [23]. The aim of the checklist is to offer guidance on the most important factors for the implementation of change in healthcare [23]. The TICD checklist was chosen as most appropriate for this study as it comprises of a comprehensive, integrated checklist of determinants of practice based on a synthesis of frameworks and taxonomies of factors that prevent or enable improvements in healthcare professional practice. The TICD can, therefore, be seen as a consensus-based checklist, built on the strengths of each of the 12 included checklists from the literature. The TICD provides a common checklist that can be used internationally across different settings and types of targeted practices to facilitate comprehensive and consistent reporting and interpretation of implementation research. The comprehensive, integrated checklist of determinants of practice (the TICD checklist) is intended as a screening tool to identify determinants that warrant further in-depth investigation. Subsequent investigation of determinants and the design of implementation strategies should focus on the factors that are most relevant for a specific recommendation. The aim of the checklist is to guide reflection and data collection on determinants of practice for a particular change, in order to explore which specific influences are most likely to be important. The idea is that this can facilitate tailoring more effective change interventions and the evaluation and reporting of tailored interventions. Additionally, it suits the patient group of interest as the checklist was developed for patients with chronic health conditions. Next to the TICD, other determinant frameworks exist, for example the Consolidated Framework for Implementation Research (CFIR), the Promoting Action on Research Implementation in Health Services (PARIHS) or the ecological framework [24]. However, none of these frameworks focus specifically on patients with chronic health conditions as the TICD does. The TICD was, therefore, deemed most appropriate for the purposes of this study. The TICD checklist consists of seven domains: (1) intervention factors, (2) individual health professional factors, (3) client factors, (4) professional interactions, (5) incentives and resources, (6) capacity for organizational change, and (7) social, political and legal factors [23]. In these seven domains possible barriers and facilitators (determinants) for implementation can be identified and, subsequently, tailored implementation strategies developed. Implementation strategies are methods or techniques to support more effective uptake of an intervention in practice [25].

The aim of this study is to identify the most important determinants for successful practice-wide implementation of the developed CCP tools for occupational and insurance physicians. Based on the results of this study, tailored implementation strategies for the implementation of CCP tools can be developed.

## Methods

In order to identify important determinants for the implementation of CCP tools, a mixed methods study with qualitative and quantitative methods was conducted. The Consolidated Criteria for Reporting Qualitative research (COREQ) checklist was used to report on the interviews and focus group process [26].

### Occupational healthcare in the Netherlands

In the Netherlands, two medical professions are crucial for occupational health: occupational physicians and insurance physicians [27]. Next to tasks in prevention of work-related health problems, in case of sick leave OPs are generally involved in the process of vocational support and return to work guidance in the first 2 years. After 2 years, insurance physicians assess whether the client is eligible for a disability benefit. In case of partial work disability, the insurance physician refers workers to a private reintegration agency. For self-employed workers, there is a possibility of self-insurance through a private insurance for work disability. However, self-employed workers are not obliged to be insured with a private work disability insurance. In the Netherlands, the Dutch occupational health services and the Dutch Social Security Institute legally fall under the Ministry of Social Affairs and Employment, whereas general and clinical care fall under the Ministry of Health, Welfare and Sport. The role of the Ministry of Social Affairs and Employment is to provide legislation on fair, healthy and safe working conditions as well as socio-economic security.

### Research setting

The study was conducted at a national level in the Netherlands as part of a larger national research consortium. The research program aimed to improve ‘worker-focused occupational health care’ and consists of four research projects. Three research projects focused on the development of evidence-based interventions (consisting of tools and trainings) to improve the supporting role of occupational physicians and insurance physicians for workers with chronic health conditions in the Netherlands. The current study is part of the fourth research project which was set up as a living lab approach and focused on further development and implementation of the semi-finished tools and trainings from the other three projects.

### Data collection

The study followed a three-step approach. Firstly, semi-structured interviews were conducted with various participants (see Table 2 for an overview of all included participants) (*N* = 21). medical specialists (cardiologist, pulmonologist, and rheumatologist) (*N* = 3), a nurse specialist (*N* = 1), insurance physicians (*N* = 2), occupational physician (*N* = 1), a client with a chronic health condition (*N* = 1), representative of a trade union for employees (*N* = 1), researchers (*N* = 6), representative of an employer’s organization (*N* = 1), general practitioner (*N* = 1), representative of a patient organization (*N* = 1), resident trainer specialized in occupational health care and insurance medicine (*N* = 2), and medical advisor private disability insurance (*N* = 1). Verbal informed consent was obtained before the start of the interview. The consent is not recorded in the transcripts as it was obtained before the start of the recording. The interviews were followed by a focus group with different experts in the field of occupational health care and insurance medicine (resident trainer (*N* = 1); occupational physician (*N* = 1); insurance physician (*N* = 2); labor expert (*N* = 1); researcher (*N* = 1); representative of a patient organization (*N* = 1)). The results of the interviews were presented per domain of the TICD. The goal of the focus group was to validate the results of the interviews. Both the interviews and focus group, were audio-recorded and transcribed verbatim. Thirdly, a survey was conducted among occupational physicians, insurance physicians, and clients with a chronic health condition with the goal to prioritize the determinants from the interviews and focus group in terms of importance. The aim of the prioritization was to set the focus for developing tailor-made implementation strategies for the implementation of the CCP tools into practice.

**Table 2:** Listing of included participants

Participant type	N
Medical specialists	
Cardiologist	1
Pulmonologist	1
Rheumatologist	1
Nurse specialist	1
Insurance physician	2
Occupational physician	1
Client with a chronic health condition	1
Representative of a trade union for employees	1
Researchers in the field of health and work	6
Representative of an employer’s organization	1
General practitioner	1
Representative of a patient organization	1
Resident trainer specialized in occupational health care and insurance medicine	2
Medical advisor of a private disability insurance	1
**Total**	21

### Recruitment of participants

Participants for the semi-structured interviews were selected through purposive sampling including persons involved in the guidance and assessment of workers with a chronic health condition. The participants were partly chosen from the supervisory committee of the research project and partly through the network of the committee. The current study was part of a larger Dutch research consortium, which engaged a wide range of stakeholders and end-users in the form of a supervisory committee in order to provide input and practical advice on the research methods and monitor the overall quality of the research project. The committee members included partners from, for example, occupational health services, the Dutch Social Security institute, private disability insurance, occupational physicians and representatives from patient organizations. Through further snowball sampling from our committee members additional participants were recruited. Participants for the focus group were recruited by means of convenience sampling by e-mail. Participants were recruited via e-mail.

### Data collection instruments

For the interviews a basic topic list was developed based on the implementation of change model [28]. The topics of the model were adapted to suit the target group of workers with a chronic health condition. Following, the topic list was supplemented by the supervisory committee of the research project. The goal was to develop a basic topic list, which can further go through an iterative development process in which the content of the list was adjusted based on the results of previous interviews. That means, for each stakeholder, topics or questions were added to fit the expertise of that stakeholder.

For the focus group interview, a topic list was developed based on the results of the interviews.

The survey was developed based on the seven domains of the TICD in co-design with representatives of the target participant groups. Co-design took place through an in-depth revision round in the form of a group meeting and written feedback by representatives of the target participant groups. This was done to ensure that all determinants per domain were included and to increase readability and usability of the survey. The survey was piloted among experts beforehand to ensure completeness and usability. The survey was self-developed based on the seven domains of the TICD and the results of the interviews and focus group. In the survey, respondents could see each domain including a description of the determinant with representative quotes. For each domain of the TICD, respondents were asked to prioritize the determinants that were identified in the interviews and focus group in order of importance (rank one = most important up to rank last = least important). Additionally, the seven domains of the TICD had to be prioritized in terms of importance at the end of the survey.

### Data analysis

The data were analyzed in three steps: 1) concept-driven coding of the interviews based on the TICD, 2) concept-driven coding of the focus group interview based on the TICD, and 3) assessment of the prioritization of the most important determinants within and between each domain of the TICD based on the self-developed survey.

The interviews and focus group were initially transcribed and analyzed by two independent researchers. The semi-structured interviews and focus group were analyzed in terms of deductive coding based on the TICD checklist. Firstly, data were coded to fit the domains of the TICD checklist and adapted to the context of occupational health. Secondly, data were deductively coded according to the adapted determinants of the TICD (Additional file 1). Coding was compared between the two researchers and any conflicts were discussed and checked by a third researcher.

The survey data were analyzed per domain by analyzing the frequency count with which a determinant ranked first in the prioritization. An a priori scenario flowchart was developed for the selection process of the most important determinants and issues resolved in the research team (Additional file 2).

Analyses of the interviews and focus group were performed in MAXQDA 2020. The survey results were analyzed in IBM SPSS Statistics 26.

### Role of the researchers and ethical considerations

The researchers (NZ, BH, AS) who analyzed the data are experienced researchers for conducting qualitative and quantitative research methods. Furthermore researcher NZ has demonstrated experience with implementation research to add to the methodological basis of this manuscript. Due to the fact that the researcher NZ, BH and AS are no occupational health experts, this might be a bias to the research results. The other authors are involved researchers in the field of occupational health and helped shape the aim and relevance of the study in the field. Data were anonymized to maintain confidentiality for the analysis. All participants gave oral informed consent to participate in the study. The study was conducted according to the guidelines laid down in the Declaration of Helsinki. The study was considered not to fall under the Dutch Medical Research Involving Human Subjects Act (WMO) as approved by the local ethics committee of the Amsterdam UMC (Reference number: W21_055#21.061).

## Results

### Interview and focus group results

The results are presented per domain and per determinant including representative quotes in Table 3. For each determinant, relevant themes were identified from the responses. For each of the determinants based on the TICD, implementation themes were identified. Examples of themes included ‘Changing routine work methods is difficult’, ‘Possibility for customization’, ‘Attention to individual’s needs and competences’, ‘Creating a sense of group-belonging among professionals’, ‘Employer must be flexible, sympathetic and cooperative’, or ‘Incentives for insurance companies’. The results of the interviews and focus group were used as the basis for developing the survey as those themes identified through the interviews and focus group served as examples for the determinants in the survey per domain.

**Table 3:** Results of the interviews and focus group

	Domain	Determinant	Theme	Quote
1.	The developed tools	How the tools were developed	Be in line with current developments in healthcare	Policy advisor Dutch Employee Insurance Agency: “…there are many developments that concern prevention and work in healthcare…. So there is now really room and opportunities to do more with it…”
			Involving industry organizations and patient associations	Resident trainer 2: “…the chance that this is successful is considerably greater if the employer has hear from the own employers’ organization that this can be of value...”
			Experiment/practice with the tools	Resident trainer 2: “…you could do pilots…you can show that its usable. That colleagues are enthusiastic about it and that patients are satisfied with it.”
		Being able to work with the tools	Enhance exposure	Resident trainer 1: “We do it mainly by putting it on our website and in our newsletters.”
			Changing routine work methods is difficult	Insurance physician 1: “Yes, [changing work method or routine] because they have been in a certain working method or routine for years.”
			Continuity of the tools after the research period	Representative of a patient organization: “Yes, so that’s very important. … That something is not developed that actually stays left alone after the research, or after the subsidy period after 4 years.”
			Do not overload professionals with new things	Insurance physician 1: “… we have to be careful … that we do not overload. … occupational and insurance physicians with this useful list, with that useful list, take this into account … and take that into account ...”
			Being in line with the current way of consultation	Representative of a private insurance: “I can imagine that when such a tool is developed, it can be added to the reporting form that we have developed over the years which is a dynamic tool.”
			Consulting at an early stage with existing education/training program	Resident trainer 2: “…at an early stage, in the co-creation phase, you should take a look to see if this [the curriculum] fits in somewhere with people from both educational specialization programs.”
			Incorporate into guidelines	General practitioner: “Well I think, it is a problem that there is still no good guideline for work, to that work-related problem.”
			Possibility for customization	Researcher 8: “It is also important that tools are not an overall, one size fits all product, … that people also have the feeling that, yes, we can shape that together in this context and in this specific situation.”
			Client version of tools/information	Researcher 8: “… we also have a very important role for patients or employers in developing care standards, and they are explicitly intended for all parties to use.”
		Necessary behavior for the use of the tools	Create awareness	Researcher 4: “It is also not always evident for them. Some say: I just don’t think about asking this during consultation.”
			Supporting guideline for the use of tools	Resident trainer 3: “… But not accepting a guidelines [for the use of tools], or acceptance around guidelines, is sometimes a problem. So that could be a threat.”
			Taking into account the background of employees	Insurance physician 2: “You should not only look at an illness, but also at the person and the system around them”
			Topic is new in resident education	Resident trainer 1: “No, in the current study program we do not really have a topic ‘Chronically ill and work’ …. But we are going to put more emphasis on this in the new curriculum.”
2.	Individual health professional factors of occupational and insurance physicians	Knowledge and skills of occupational and insurance physicians	Professionals are already familiar with the topic	Insurance physician 1: “… there is already a considerable level of expertise. It’s not that you have to teach them something new.”
		Cognitions, beliefs and attitudes of occupational and insurance physicians	Professionals find the topic important	Insurance physician 1: “… many professionals recognize this [the importance of tools] and also see this as something that helps them to do their work better.”
			Show added-value	Medical advisor private disability insurance: “… showing them [occupational and insurance physicians] how much added-value, or how attractive it is, to use it. If an occupational health expert is greatly supported by such a tool to get a clearer picture of, for example, those environmental factors, then, I think, he will use it…”
			Conviction that the tool will work	Resident trainer 2: “Much remains in the idea of ‘I am not yet convinced that this is easy for me and that it has added value’. … Then things remain unused.”
			Self-confidence in own ability	Resident trainer 3: “I think, that confidence in one’s own abilities could be strengthened, especially by paying attention to it in training and education.”
			Belief that the chronically ill worker is an expert	Researcher 8: “…the belief that the chronically ill worker is also an expert on this topic, and the expertise you need to involve in that. That’s also a very important cognition.”
		Professional behavior of occupational and insurance physicians	Lack of time	Researcher 4: “Yes, but what I hear from the occupational physicians is, that they are much too busy. They are very much swamped in work.”
3.	Client factors of workers with a chronic health condition	Client needs	Attention to individual needs and competences	Representative of a patient organization: “… They mainly look at how people can work 40 h a week instead of really looking at the core, the competences of someone and, that maybe it’s better for such a person to work 4 h a day ...”
			Practical and concrete information	Representative of a patient organization: “For us it really has to result in something concrete with which we can actually keep chronically ill people better in their jobs.”
			Possibility to try what works (for client)	Client with a chronic health condition: “… I also always tried, together with the occupational physician … to see: ok, what can I do, what can’t I do? What is a good distribution of working hours? … a bit of trial and error of seeing what works.”
			More attention for work from patient organizations	Resident trainer 2: “It could … help that … patient organization around the chronically ill refer information about work support options to their patients.”
			Stress and strain	Representative of a patient organization: “… the moment you develop a condition [chronic health condition], that’s often a lot [for a person]. It also often means that you are looking for ways on how you will organize your daily life, and also your work. A lot of uncertainty: ‘what’s going to happen to me?’ A lot of stress for people.”
			Self-employed person no occupational physician	Medical specialist 1: “… they are … now more and more self-employed and of course they do not have an occupational physician yet. … This is really a barrier.”
		Client beliefs and knowledge	Awareness	Insurance physician 2: “And, I think, that it has to do with awareness when people maintain their own situation, because they do not want to [change]. And moving from not wanting to wanting to change requires a lot of time and energy.”
			Knowledge about (preventive) role of an occupational physician	Researcher 5: “… the occupational physician also has a preventive task. But … in practice, they are mainly known for their guidance concerning absenteeism and return to work.”
			Skills in communication, self-management is assumed	Resident trainer 1: “… the self-sustainability model, … is a great model. But, … a lot of people cannot do it [self-sustainability]. … they have limited health skills, limited coping.”
			Negative image of occupational/insurance physician	Researcher 4: “In general, I don’t think the occupational physician has a very positive image.”
			There is already a lot of information available for chronically ill employee, but it is often scattered and difficult to find	Representative of a patient organization: “There is a lot of information, but not findable. So, that is actually the very first step of making what there is findable.”
			Flawed information provision	Researcher 2: “What has also been identified is that the workers have little information about the entire procedure for disability assessment ....”
		Client preferences	(Trust) relationship between occupational physician and employer with a chronic health condition	Representative of a trade union for employees: “I think, that an occupational physician that the employee can trust is very important.”
		Client motivation	Contributing to society	Worker with a chronic health condition: “Yes, … what motivates is simple: I want to participate in society. I don’t want to sit at home.”
			Attain identity with work	Researcher 6: “… while I also had someone who said: ‘my job is just who I am.’ … Or well, not your entire identity, but a large part.”
			Motivation/being motivated	Nurse specialist: “… Yes, how motivated someone is to adjust his lifestyle. And those are the conversations you also have with people.”
			(Job) insecurity and vulnerability	Resident trainer 2: “… Someone who is chronically ill or elderly and has a chronic background that makes applying for a job seem hopeless, [that person] is not motivated to apply.”
			Finding work less important after falling ill	Researcher 6: “Some people see their work as less important after their illness.”
			Making time for treatment (no work means no income for self-employed people)	Medical specialist 2: “People who work, the younger population, do not have time for the outpatient clinic visit, because they have to work. … They are self-employed and no work means no income.”
		Client behavior	Keep in touch with employer	Nurse specialist: “… if people feel heard, then I have the idea that they are less likely to report sick. That they feel involved in the company.”
			Show initiative/take action/get moving as an employee with a chronic health condition	Worker with a chronic health condition: “… self-management is more in the sense that you keep the responsibility of what happens to you at work and with your workplace, for example. … And that is not decided without your consent for you by your employer or the occupational physician.”
			Stay positive	Worker with a chronic health condition: “… So in that respect, like me, he has always tried to see the positive side: ‘look, what you can still do.’.
			Be transparent	Representative of a trade union for employees: “… it is, of course, important to get a good picture of the exact effects on work of that chronic health condition. And what someone needs in order to function properly. What is possible and what is not possible. And what you have to take into account.”
4.	Professional interactions	The influence of beliefs, ideas and communication between healthcare professionals	Mutual trust between occupational physicians and curative physicians	Researcher 6: “And the internist indicated that he actually did not have that much confidence in the knowledge and medical knowledge of the occupational physicians.”
			Protective advice with regard to work in curative care	Medical advisor private disability insurance: “… it may be that the practitioners have a very protective influence and actually do not want patients to meet the challenge of returning to work.”
			Personal interest of physician	Occupational physician 1: “It also varies per specialist. Some are more open than others. Sometimes I see people directly, where the specialist said: ‘also go and talk to your occupational physician’.”
		Teamwork between professionals	Much involvement in guidance by the health and safety service	Resident trainer 2: “… in practice, you often see that other people also do that consultation hour … guidelines are still based on the idea that there is a single physician who acts while it is different in practice.”
			Little or no feedback on the actions of the insurance physician	Insurance physician 2: “… what I find a pity, also for our clients, is, that you will never actually get to hear how it continues with them.”
			Medical specialist at consultation hour with occupational physicians	Insurance physician 2: “I do not rule out that we go to situation that an insurance physician is present in the hospital, who does consultation hours and/or gives advice to medical specialist colleagues …”
			Creating a sense of group-belonging among professionals	Resident trainer 2: “… the most important part of training is not so much the transfer of knowledge. It is the collective idea of: ‘we are going to do something with this’.”
		Coordination and collaboration between healthcare professionals	Attention towards work in curative care	Occupational physician 1: “That is also a general problem, the focus on work within curative care. ... But that should, … become almost normal for the various specialists.”
			Thinking from a medical model/perspective	Resident trainer 1: “As doctors, we often tend to focus on the disease. The fact that someone has a medical condition also asks for focus on functioning, but also: ‘what is hindering him?’. … The context often determines someone’s ability to function and feeling healthy more than anything else.”
			Inadequate information transfer and exchange	Medical specialist 1: “Never before has an occupational physician called me. Maybe it’s not allowed, I don’t know. I don’t think consultation with each other would hurt.”
			Knowledge about work in curative care	Occupational physician 1: “That the healthcare professional says: ‘also talk to your occupational physician´. If patients are unfamiliar with occupational healthcare, that a treating physician knows occupational medicine, then you [occupational physician] can also give tips and discuss what will help.”
			Medical knowledge and working method of occupational and insurance physician	Insurance physician 2: “… I think, it [occupational health] is a separate kind of field. … what you should have, … is the interest in the client and in what such an illness means for someone and how you could get someone out of the vicious circle of incapacity.”
			Experienced hierarchy between medical specialists	General practitioner: “You also have a sort of hierarchy ranking where cardio-thoracic surgery and neurosurgery are high up in the hierarchy … GPs are somewhere in between … and occupational physicians are lower ranked. That also, partly, determines the willingness to cooperate.”
			Make use of task delegation	Resident trainer 3: “I think, in this domain, a lot of work is done together with other disciplines.”
			Common/shared interest with involved stakeholders	Resident trainer 3: “… point incentives and means in the same direction. Do not work against each other.”
5.	Incentives and resources	Availability of required resources	Need for a good instrument	Occupational physician 1: “Especially If you have it [digital screening support] for self-management or self-control, a kind of questionnaire that quickly shows how someone is dealing with it. Of course, we [occupational health services] are also increasingly moving towards digital questionnaires to fill in before people [workers with a chronic condition] come to consultation hours.”
		Positive and negative financial incentives	Continuity of the company in the event of an employee’s illness	Representative of an employer’s organization: “Where big and small companies differ in continuity of business operations is, that large companies have the scale to do more in the sense of guidance and discontinuity problems …”
		Non-financial positive and negative incentives	Employer invests in a good employee	Representative of a trade union for employees: “… if an employer is satisfied with an employee who has a chronic health condition, … then most employers are likely to make required work adjustments.”
			Have confidence in the employee with a chronic health condition and take it seriously	Representative of a patient organization: “I also hear stories from people who have complaints, that they do not feel understood by the occupational physician and, that they think the occupational physician is too much on the side of the employer.”
			Adding work to programs/teams in the curative sector	Medical specialist 1: “The cardiac rehabilitation tool. That program runs very well. So you can easily merge them.”
			Corporate culture	Representative of a trade union for employees: “…it all depends on the type of employer and the culture in the company. Much more [change towards work improvement] is possible in one company than in another.”
		Information systems	Join with existing IT systems	Resident trainer 2: “… it could be useful if something can be included in the software that we already use to write consultation hour reports.”
		Quality and patient safety	Work as an outcome measures for the inspectorate and registration	Medical specialist 1: “You have to make it compulsory. It [work-related support] just has to become one of the outcome measures of the inspectorate.”
		Refresher course training systems	Accreditation points for re-registration	Resident trainer 1: “But, you just need 40 points a year to re-register for 5 years. So, if necessary, you choose a training that yields points.”
			Work more embedded in basic medical training	Medical specialist 1: “I think, that work and health are so intertwined. … that it is good that we pay lot of attention to this in all phases of medical training. Also if you become a medical specialist.”
		Presence of practical tools for healthcare professionals	No standardized tool is available yet	Researcher 2: “… occupational and insurance physicians in any case did not indicate that they already use a tool [to support work-related issues] structurally.”
6.	Capacity for change in organizations/by employers	Mandate and authority	Employer has responsibility	Occupational physician 1: “For someone who works with a partial disability benefit for an employer, the employer also has the responsibility to see how someone is doing.”
		Skilled leadership	Open and communicative atmosphere in the workplace	Representative of an employer’s organization: “Employers need that knowledge to know what to ask of people [employees with a chronic health condition].”
		Strength of support and opposition	Employer must be flexible, sympathetic and cooperative	Resident trainer 1: “… it also depends on the employers. How willing is an employer to keep someone with a chronic health condition employed and give them the opportunity to work.”
			Costs associated with an employer with a chronic health condition/continued payment in the event of illness	Representative for a trade union for employees: “So, we look at: what is good for the company? And what, in our opinion, is the best approach to have employees working as much as possible? Because, yes, we pay for productive employees, and sick employees or employees who do not perform well. They cost money instead of making money.”
			Express appreciation	Representative of a trade union for employees: “The employer’s appreciation for the employee. So, a kind of mutual appreciation, reciprocity, is, I think, a very important precondition moving forward.”
			Acceptance and inclusivity at the workplace	Researcher 6: “That they [workers with a chronic health condition] are accepted and that we also want to keep you working. … you are important to the company.”
		Regulations, rules, policy at employers/organizations	Occupational health services are commercially competitive	Resident trainer 2: “… there is no uniformity [in occupational health services], because the government has decided that it [the occupational health service] is a commercial and competitive service.”
		Priority of desired change	Employer must be open to prevention	Occupational physician 1: “When a workers needs it, you can go for a preventive consultation. We all have to work much more towards prevention. Even, if you already have a chronic health condition ….”
			Perception that the employee with a chronic health condition is difficult/expensive for the employer	Resident trainer 1: “I think that in many work places there is less room for people with disabilities, because employers think: ‘Yes, this person is difficult’.”
			Demanding attention for topics at the occupational health services	Resident trainer 2: “… you can distinguish yourself in the market, if you pay attention to this [specific support needs for workers with chronic health conditions] as an occupational health service.”
			Appropriate workplace adaptations	Representative of an employer’s organization: “It is important that the employee can return to work within the company as good as possible. Just integrate well into the company, as in return to his old position.”
			Having knowledge of sick leave rights and obligations	General practitioner: “I notice, that many GPs, they advise patients to contact the occupational physician …. Yesterday, a patient who had a heart attack relatively recently showed up at his boss who asked: ‘when can you start again’. … He could use the protection from the occupational physician very well.”
		Monitoring and feedback	Discontinuity in guidance of workers with a chronic health condition by the occupational health service	Occupational physician 1: “It occurs that I guide a sick-listed worker and then, due to change of occupational health services, I have to transfer him to a colleague, who also needs to get to know the worker and study the worker’s medical records.”
		Help needed with change in organizations	Take the burden from the employer	Representative of an employer’s organization: “It is also important to minimize the burden for the employer. Ensure that it does not become an administrative hassle. That’s a basic requirement.”
			Tailor-made advice for the employer	Representative of a trade union for employees: “.. an employer would prefer an occupational health service and occupational physician who takes the company and the interests of the company into account as much as possible.”
			Information is applicable and implementable	Representative of an employer’s organization: “Information provision, not only to give dry, technical information, but also to give the information that can be directly applied, … aimed at ‘doing’.”
			Practical and solution-oriented information	Representative of an employer’s organization: “… employers never ask for answers. They ask for solutions. … how a problem can be solved, and how he can continue to supply his product or continue to provide his service.”
7.	Social, political and legal aspects	Contracts	Major differences between (contracts with) occupational health services	Occupational physician 1: “Yes, there is no uniformity, because the government has decided that it is a commercial and competitive service. As in every market, there a different needs and wishes and employers can look at a price and quality differences and buy something that they think matches to what they want.”
			Many changes in occupational physicians within occupational health services	Representative of a trade union for employees: “… the problem is that employers can replace the occupational health service and occupational physician at any time, for whatever reason.”
		Legislation	Curative care and occupational health are strictly separated	Medical specialist 2: “I don’t know who the occupational physician is … I never actually get information form the occupational physician.”
			Insufficient support for employers	Representative of an employer’s organization: “… a mismatch between the functional abilities of an employee [with a chronic health condition] and the job requirements can be challenging for an employer. First an employer needs to know what appropriate workplace adjustments are, next application and then it takes time to realize these adjustments, that is costly. These things don’t help.”
			Unknown with regulations, rights and obligations	Medical specialist 3: “Look, you also have people who work at a small company, who say: ‘Do we have an occupational physician, I would not know’. Or they say: ‘my supervisor does not allow me to go there’. So, I always say that you just have to, you are entitled to that.”
		Financing policy	Incentives for insurance companies	Representative of a patient organization: “The disability insurance company, so, they have to pay premiums when people drop out. To see if they [the disability insurance company] can, for example, give incentives to companies that make these tools accessible to their HR department. … get a discount on the premium, or something like that.”
			No reimbursement for work-related healthcare in the curative sector	Medical specialist 1: “It [the current system] makes it difficult to get reimbursement for work-related healthcare. What I also thought of is that you could make a kind of outpatient clinic for work. But that, of course, does not fall under reimbursed care. So, that is for us, I think a pity, that we cannot just do an occupational health consultation. What we can do for all other medical specialisms.”
			Financing policy municipalities	Insurance physician 2: “… that is municipal policy. … yes, the municipality usually wants to be in the first place for saving money. So, actually, they want to have research as good as possible and as cheap as possible.”
			Public funding of work-related care is needed	Occupational physician 1: “Work-related care within primary and secondary care is actually not covered. You could say let the employers pay for all of that. I think, that is not always entirely realistic. Certainly for people with a chronic health condition, work-related care should partly fall under reimbursed care.”
		Influential people	Deploy role models	Resident trainer 2: “Physicians look very closely at whether someone of their own kind gives a positive advice.”
		Political stability	Influence of political decision-making	Insurance physician 2: “… they really want something [a relation] with the client, but it is not always clear what the underlying motive is. Sometimes it is really just the political pressure to guide people [clients with a chronic health condition].”

### Survey results with representative quotes from the qualitative analysis

**Fig. 1:**
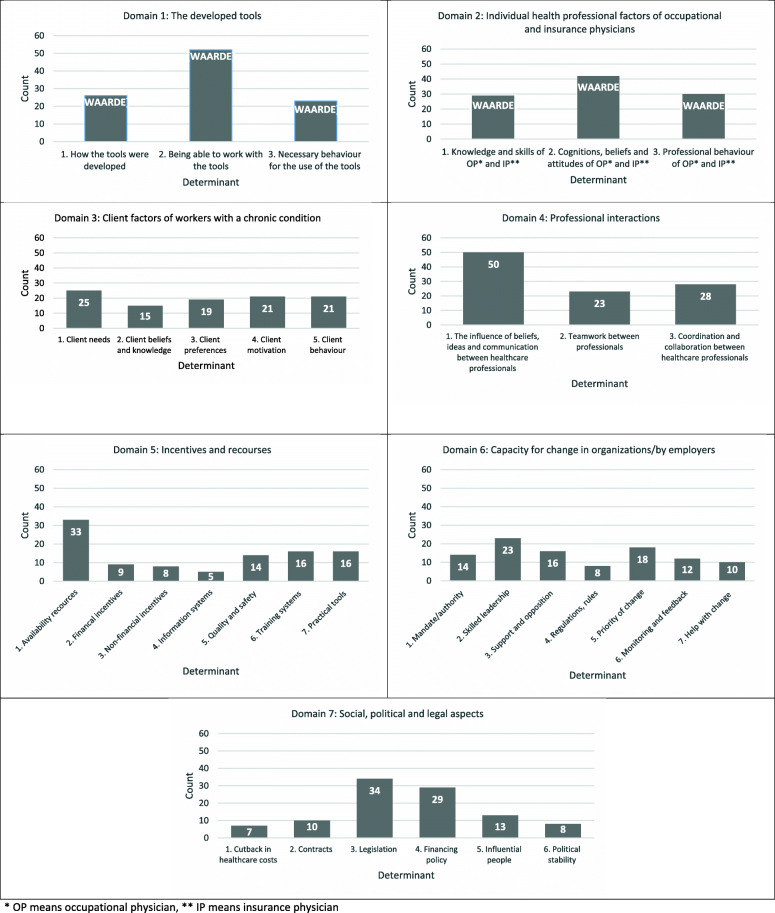
Prioritization of determinants per domain for the seven domains of the TICD. * OP means occupational physician, ** IP means insurance physician. The figure describes the frequency results per TICD domain

A total of 101 respondents participated in the survey. Table 4 shows the characteristics of respondents. Most respondents were either OP (*N* = 56) or IP (*N* = 40). The results of the prioritization per domain are presented in Fig. 1. For each domain the determinant that ranked as most important is presented and followed by an illustration based on themes as facilitators and barriers for successful practice-wide implementation.

**Table 4:** Participant characteristics prioritization survey

Characteristic	Survey data (***N*** = 101)
Men, n (%)	53 (52.5)
Women, n (%)	48 (47.5)
Age, mean (SD)	55.5 (9.7)
Stakeholder group	
Occupational physician, n	56
Insurance physician, n	40
Worker (with a chronic health condition), n	6
Other^a^, n	8

^a^Other included: insurance physician in training, occupational physician in training, reintegration coach, general practitioner, clinical occupational expert, patient with a disability

#### Domain 1: the developed tools

The determinant that ranked most important was ‘being able to work with the tools’ (*N* = 52) in this domain. This determinant concerns practical questions concerning the use of the tools for the occupational or insurance physician or the employer, for example in terms of time and usability. In order to be able to work with the tools, the respondents, for example, mentioned the following considerations as facilitators for the implementation: “enhance exposure” which includes efficient findability; “incorporate into guidelines” and “possibility for customization”. One respondent said:

*“It is also important that tools are not an overall, one size fits all product, … that people also have the feeling that, yes, we can shape that together in this context and in this specific situation.” (Researcher 8)*

As an important barrier it was mentioned that it is difficult for physicians to change their current way of working:

*“Yes, [changing work method or routine] because they have been in a certain working method or routine for years.” (Insurance physician 1)*

#### Domain 2: individual health professional factors of occupational and insurance physicians

The majority of respondents ranked ‘cognitions, beliefs and attitudes of occupational and insurance physicians’ as the most important factor in this domain (*N* = 42). Establishing a need for the tools for improving the current way of working was mentioned as a facilitator:

*“… showing them [occupational and insurance physicians] how much added-value, or how attractive it is, to use it. If an occupational health expert is greatly supported by such a tool to get a clearer picture of, for example, those environmental factors, then, I think, he will use it…” (Medical advisor private disability insurance)*

Respondents mentioned that professionals also need to believe in their own competences and abilities to be able to use the tools:

*“I think, that confidence in one’s own abilities could be strengthened, especially by paying attention to it in training and education.” (Resident trainer 3)*

#### Domain 3: client factors of workers with a chronic health condition

Most of the respondents ranked ‘client needs’ as the most important determinant (*N* = 25) in this domain. Respondents indicated that when developing a tool to support a worker with a chronic health condition, it is important to consider the individual needs and wishes of the patient/client:

*“They mainly look at how people can work 40 hours a week instead of really looking at the core, the competences of someone and, that maybe it’s better for such a person to work four hours a day ...” (Representative of a patient organization)*

A limiting factor for successful use and implementation of the tools could be stress of the clients:

*“… the moment you develop a condition [chronic health condition], that’s often a lot [for a person]. It also often means that you are looking for ways on how you will organize your daily life, and also your work. A lot of uncertainty: ‘what’s going to happen to me?’ A lot of stress for people.” (Representative of a patient organization)*

Although the result for this domain ‘client needs’ was conclusive, it ranked close to ‘client motivation’ and ‘client behavior’ as second-most important determinants which were ranked by an equal amount of respondents (*N* = 21). Workers feel the need to contribute to society:

*“Yes,… what motivates is simple: I want to participate in society. I don’t want to sit at home.” (Worker with a chronic health condition)*

Moreover, workers experience a sense of taking own responsibility:

*“… self-management is more in the sense that you keep the responsibility of what happens to you at work and with your workplace, for example. … And that is not decided without your consent for you by your employer or the occupational physician.” (Worker with a chronic health condition)*

#### Domain 4: professional interactions

The respondents chose the determinant ‘the influence of beliefs, ideas and communication between healthcare professionals’ as most important (*N* = 50) in this domain. Respondents pointed out that what healthcare professionals know and think about each other’s work is an important factor in collaboration and communication:

*“And the internist [specialist for internal diseases] indicated that he actually did not have that much confidence in the knowledge and medical knowledge of the occupational physicians.” (Researcher 6)*

It was mentioned that communication and collaboration may also vary between specialists, which could hinder the collaboration with Ops and IPs:

*“It also varies per [medical] specialist. Some are more open than others. Sometimes I see people directly, where the specialist said: ‘also go and talk to your occupational physician’.” (Occupational physician 1)*

#### Domain 5: incentives and resources

The determinant ‘availability of required resources’ was chosen as the most important determinant among the seven determinants in this domain (*N* = 33). The extent to which resources are available, such as technical equipment or IT support was mentioned by the respondents:

*“Especially If you have it [digital screening support] for self-management or self-control, a kind of questionnaire that quickly shows how someone is dealing with it. Of course, we [occupational health services] are also increasingly moving towards digital questionnaires to fill in before people [workers with a chronic condition] come to consultation hours.” (Occupational physician 1)*

#### Domain 6: capacity for organizational change (in organization or by employer)

For this domain the determinant ‘skilled leadership’ was ranked as most important (*N* = 23). It was indicated that the employer should have a responsibility to support and check on their employee in terms of suitable workplace adjustments. In order to do so, a certain open and communicative culture is important:

*“Employers need that knowledge to know what to ask of people [employees with a chronic health condition].” (Resident trainer 1)*

Even though this determinant was evidently ranked as most important, it was closely followed by ‘priority of desired change’ (*N* = 18) in this domain. The respondents indicated that employers’ may perceive a worker with a chronic health condition as difficult and expensive:

*“I think that in many work places there is less room for people with disabilities, because employers think: ‘Yes, this person is difficult’.” (Resident trainer 1)*

#### Domain 7: social, political and legal factors

The determinant ‘legislation’ ranked as most important (*N* = 34) in this domain. The strict separation of both the Dutch health ministry and the Ministry of Social Affairs and Employment also influences information flow between the clinical care sector and occupational health practitioners as well as insufficient support for employers:

*“… a mismatch between the functional abilities of an employee [with a chronic health condition] and the job requirements can be challenging for an employer. First an employer needs to know what appropriate workplace adjustments are, next application and then it takes time to realize these adjustments, that is costly. These things don't help.” (Representative of an employer’s organization)*

#### Overall prioritization of the most important domain

**Fig. 2:**
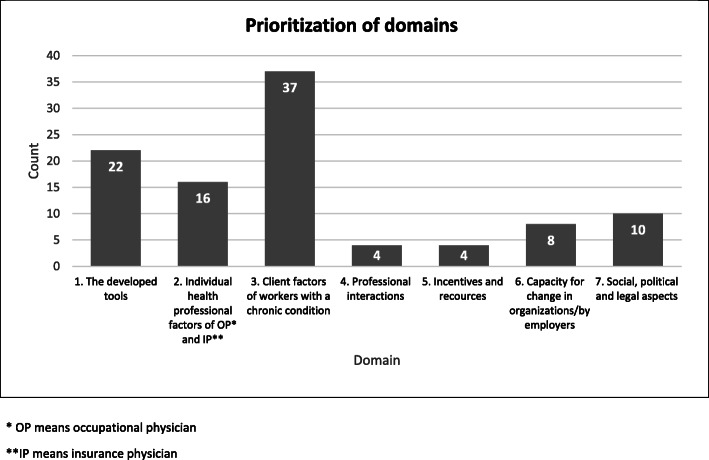
Prioritization of TICD domains. * OP means occupational physician, **IP means insurance physician. The figure presents the overall prioritization frequency count of the TICD domains

At the end of the survey, respondents were asked to rank the seven domains according to importance. The respondents ranked domain 1: ‘client factors of workers with a chronic health condition’ as the most important domain for successful practice-wide implementation of the CCP tools (Fig. 2).

## Discussion

This study identified the most important determinants for successful practice-wide implementation of CCP tools. For this purpose, the TICD was used as a framework to guide the analysis. The domain that ranked highest was "client factors associated with workers with a chronic health condition". Within this domain clients’ needs were identified as most important factor for successful implementation of the CCP tools. The needs of clients including individual person’s needs and competences, accurate and specific information, increased attention for work circumstances from patient organizations, being aware of stress of clients that is associated with dealing with a chronic health condition, the possibility to discuss options with the professional concerning work participation or their ability to work and being aware of the situation that self-employed workers often do not have access to an occupational physician were reflected on by participants in the interviews and focus group. Most of the self-employed workers are not entitled to social benefits when they are short of work, ill, or disabled. This also means no access to occupational health services and support. Self-employed individuals in the Netherlands can voluntarily insure themselves against sickness absence and long term work disability and receive benefits and occupational health guidance when falling ill or sustaining injury and hereby experiencing health-related work disability. However, for many Dutch self-employed workers private insurance is too costly, resulting in four out of 10 self-employed workers without disability insurance and no access to occupational healthcare [29].

### Implications of study findings in context of existing research

#### Workers with chronic health conditions

For workers with chronic health conditions, taking into account their needs was found paramount for the implementation of CCP tools. Attention towards individual’s needs lies at the heart of client factors. An earlier study identified the understanding of what is possibly at the core of CCP [7]. Only by understanding the client’s needs, the client can really become the focal point of occupational health services. The developed tools specifically focus on improved understanding of the client’s needs, their cognitions and perceptions and their social environment or context. Development and implementation of the tools while taking into account the clients’ needs is a prerequisite to the feasibility and effectiveness of these tools which is also confirmed in an earlier systematic review [30]. In the current study, workers with chronic conditions and various stakeholders involved in supporting and guiding work participation participated in co-creation. By involving the end-users the needs of clients can be understood and can be taken into account.

#### Using a systematic framework for the identification of important implementation factors

The current study, did not only take into account the necessary domains of the TICD checklist, but also evaluated determinants for implementation within each domain. Most studies concerning interventions for workers with a chronic health condition focus on the effectiveness evaluation of the intervention itself but pay no or little attention to the dissemination and implementation into practice [31, 32]. In contrast to effectiveness studies, the current study focused on a wide array of contextual factors that may influence implementation and supports shaping concrete implementation strategies with a holistic approach using the TICD. As the TICD focuses on chronic diseases it was certainly suitable for this study as the focus for the CCP tools are clients with chronic health conditions. The TICD has also been used in other health care settings and proved valuable which furthermore strengthens the possibilities for application of the TICD domains within occupational health practice as it presented to be applicable in a versatile manner [33]. Hence, by using the TICD in our study we were able to consider all levels needed for the identification of implementation factors.

#### Implementation in occupational health care practice

The implementation of person-centered tools for workers with chronic health conditions is complex and dependent on several important determinants [34]. The importance to take contextual factors into account when implementing changes into health care practice has been recognized earlier [34]. The goal of the current study was, therefore, to identify the most important determinants for implementation by looking at a wide array of domains and determinants from the TICD. The literature describes contexts in implementation research as dynamic since barriers in one environment may be facilitators in another [35]. Given that occupational health is a complex, multi-level system with numerous factors that may affect successful implementation, it is imperative to obtain a broad and qualitative insight into the broad array of relevant implementation factors, which emphasizes the need to identify the most important determinants as done in our study [36, 37]. Earlier studies identified individual’s engagement, beliefs and knowledge with regard to the intervention, lack of integration with organizational processes, commitment of management, allocation of financial resources and training as significant determinants for implementation [21, 36]. These determinants were also found in our study. But the highest ranked factor in our study, acknowledging the clients’ needs, was also found imperative for successful implementation of interventions with focus on chronic health conditions in a different study [38]. Furthermore, access to appropriate information in an easily understandable way and support and availability of healthcare providers were found of influence for the implementation [38]. However, in our study, the themes that emerged from the determinant ‘client’ needs were rather focused on personalization and tailoring of approaches. An earlier systematic review identified that patient level measures were least likely to be assessed in implementation studies [39], but it was highlighted that it is paramount to assess appropriateness and feasibility with the patient population before implementation [39]. The results of the current study can, therefore, serve as the starting point for practice-wide implementation by taking into account the clients’ needs. The results of our study form the basis for the design of tailored implementation strategies focused on the clients’ needs.

### Strengths

A strength of the current study is the methodological approach with a mixed-method design in three steps, namely interviews, focus group and quantitative survey data. The application of mixed-method study designs can enhance understanding of the research question studied [40]. By interviewing participants, in-depth perspectives from individuals and the group were examined. The survey data, on the other hand, helped prioritize the wide range of results from the individual interviews and focus group, which supports setting focus for implementation strategies in practice. Another strength is the involvement of various stakeholders involved in occupational health, as well as clients with a chronic health condition. By involving the end-users of the CCP tools and clients, co-creation was guaranteed. Co-creation can enhance involvement of stakeholders in co-creating value, enhance new innovation developments, strengthen network solutions and may enhance an entire service system [41]. Moreover, by using the TICD checklist a wide spectrum of implementation factors was considered, which could also be of added-value to the implementation of other tools into practice.

### Limitations

Despite the strong methodological approach with data triangulation, the study faced several limitations. Firstly, this study presents important determinants for the implementation of CCP tools for workers with a chronic health condition in the Netherlands and may, therefore, lack generalizability. Therefore, the results should be considered in light of the Dutch context. The determinant that scored as most important, however, was client factors, which is meaningful in different contextual environments. Secondly, the survey was mostly completed by OPs and IPs. We attempted to include the perspective of the employers and workers, but only reached limited response of workers with a chronic health condition due to the covid-19 crisis, which restricted questionnaires to be sent by a patient organization and a trade union for employees to prevent overload due to other covid-19 -related questionnaires that were already shared. Even though a large health and safety service was involved in the dissemination of the survey, we did not acquire responses as the questionnaire was also not a priority due to the covid-19 measures at that time. This could, therefore, have led to bias in the selection of the most important domain and determinant. Thirdly, due to feasibility, the goal of the current study was to choose the most important determinants. This choice does not necessarily mean that the other domains and determinants should be neglected, but rather sets focus for developing targeted and feasible implementation strategies for the implementation of the presented CCP tools.

### Implications for future research

To the best of our knowledge, this is the first study assessing determinants for the implementation of CCP tools for workers with chronic health conditions in occupational health practice using the TICD checklist. Based on the TICD checklist, we were able to take into account all relevant domains for the implementation which might not have appeared without the guidance of the TICD. The TICD checklist, though helpful in designing an implementation study and facilitating consistent reporting of implementation research, it required further in-depth investigation of the identified implementation determinants from the interviews and focus group. In order to explore which determinants were perceived as most important by the various stakeholders, a prioritization survey was developed. Future studies concerning clients with chronic health conditions may also apply the TICD checklist for guidance, but should be aware that unless a wide range of determinants are desired, additional quantitative or additional rounds of interviews should be conducted to set focus on the most important determinant(s). Ultimately, in order to establish strategies for successful implementation of tools into practice, it is important to identify the most critical determinants that may enable or impede practice-wide implementation. Occupational health is a unique and dynamic system that requires detailed implementation support. Therefore, future studies with the aim of practice-wide implementation should also focus on detailed assessment of determinants for the implementation. The process, however, may be quite lavish as preferably various stakeholders are involved. Therefore, less demanding methods for assessing key implementation determinants may be considered in future studies.

## Conclusion

The present study identified the most important determinants from the perspective of various stakeholders involved in the implementation of CCP tools in occupational health for workers with chronic health conditions. Based on the identification of the most important determinants, targeted implementation strategies can be developed to support successful practice-wide implementation of CCP tools.

## Supplementary Information

**Additional file 1.** Overview of the TICD domains and determinants adapted from Flottorp et al. (2013). Data from the TICD have been adapted for the research context of occupational health.

**Additional file 2.** Scenario Flowchart data analysis prioritization determinants based on survey data. The appendix describes a flowchart for the decision on the most important determinant per domain of the TICD.

## Data Availability

The datasets used and analyzed during the current study are available from the corresponding author on reasonable request.

## Abbreviations

PCC: Person-centered care
CCP: Client-centered practice
TICD: Tailored Implementation for Chronic Diseases
OPs: Occupational physicians
IPs: Insurance physicians
COREQ: Consolidated Criteria for Reporting Qualitative research
CFIR: Consolidated Framework for Implementation Research
PARIHS: Promoting Action on Research Implementation in Health Services
