# Supplementary material for: Determinants for the implementation of person-centered tools for workers with chronic health conditions: a mixed-method study using the Tailored Implementation for Chronic Diseases checklist
Source: BMC Public Health. 2021 Jun 7;21:1091. doi: 10.1186/s12889-021-11047-6 (PMC8183322; doi:10.1186/s12889-021-11047-6)
Supplement: Supplementary file 1 — Additional file 1. Overview of the TICD domains and determinants adapted from Flottorp et al. (2013). Data from the TICD have been adapted for the research context of occupational health. [file 12889_2021_11047_MOESM1_ESM.docx]

**Determinants for the implementation of person-centered tools for workers with chronic health conditions: a mixed-method study using the tailored implementation for chronic conditions framework.**

Zipfel, N.^1^, Horreh, B.^1^, Hulshof, C.T.J.^1^, Suman, A.^1 2^, de Boer, A.G.E.M.^1^, van der Burg-Vermeulen, S.J.^1^

1. Amsterdam University Medical Centers, University of Amsterdam, Department of Public and Occupational Health, Coronel Institute of Occupational Health, Amsterdam Public Health research institute, PO Box 22700, 1100 DE Amsterdam, The Netherlands
2. Julius Center for Health Sciences and Primary Care, University Medical Center Utrecht, Utrecht University, Utrecht, The Netherlands

Corresponding author: N. Zipfel, n.zipfel@amsterdamumc.nl

Additional file 1. Overview of the TICD domains and determinants adapted from Flottorp et al. (2013).

| Number | Domain | Determinant |
| --- | --- | --- |
| 1. | The developed tools | How the tools were developed |
|  |  |  |
|  |  |  |
|  |  | Being able to work with the tools |
|  |  |  |
|  |  |  |
|  |  |  |
|  |  |  |
|  |  |  |
|  |  |  |
|  |  |  |
|  |  |  |
|  |  | Necessary behaviour for the use of the tools |
|  |  |  |
|  |  |  |
|  |  |  |
| 2. | Individual health professional factors of occupational and insurance physicians | Knowledge and skills of occupational and insurance physicians |
|  |  |  |
|  |  | Cognitions, beliefs and attitudes of occupational and insurance physicians |
|  |  |  |
|  |  |  |
|  |  |  |
|  |  |  |
|  |  | Professional behaviour of occupational and insurance physicians |
| 3. | Client factors of workers with a chronic condition | Client needs |
|  |  |  |
|  |  |  |
|  |  |  |
|  |  |  |
|  |  |  |
|  |  |  |
|  |  | Client beliefs and knowledge |
|  |  |  |
|  |  |  |
|  |  |  |
|  |  |  |
|  |  |  |
|  |  | Client preferences |
|  |  | Client motivation |
|  |  |  |
|  |  |  |
|  |  |  |
|  |  |  |
|  |  |  |
|  |  |  |
|  |  | Client behaviour |
|  |  |  |
|  |  |  |
|  |  |  |
| 4. | Professional interactions | The influence of beliefs, ideas and communication between healthcare professionals |
|  |  |  |
|  |  |  |
|  |  | Teamwork between professionals |
|  |  |  |
|  |  |  |
|  |  |  |
|  |  | Coordination and collaboration between healthcare professionals |
|  |  |  |
|  |  |  |
|  |  |  |
|  |  |  |
|  |  |  |
|  |  |  |
|  |  |  |
|  |  |  |
| 5. | Incentives and resources | Availability of necessary resources |
|  |  | Financial incentives and disincentives |
|  |  | Nonfinancial incentives and disincentives |
|  |  |  |
|  |  |  |
|  |  |  |
|  |  |  |
|  |  | Information system |
|  |  | Quality assurance and client safety systems |
|  |  | Continuing education system |
|  |  |  |
|  |  | Presence of practical tools for healthcare professionals |
| 6. | Capacity for organisational change (in organisation or by employer) | Mandate, authority, accountability |
|  |  | Capable leadership |
|  |  | Relative strength of supporters and opponents |
|  |  | Regulations, rules, policies from organisations/employers |
|  |  | Priority of necessary change |
|  |  | Monitoring and feedback |
|  |  | Assistance for organisational changes |
| 7. | Social, political and legal factors | Cutbacks in healthcare costs |
|  |  |  |
|  |  | Contracts |
|  |  |  |
|  |  |  |
|  |  | Legislation |
|  |  |  |
|  |  |  |
|  |  | Payer or funder policies |
|  |  |  |
|  |  | Influential people |
|  |  | Political stability |
