# Supplementary material for: Determinants for the implementation of person-centered tools for workers with chronic health conditions: a mixed-method study using the Tailored Implementation for Chronic Diseases checklist
Source: BMC Public Health. 2021 Jun 7;21:1091. doi: 10.1186/s12889-021-11047-6 (PMC8183322; doi:10.1186/s12889-021-11047-6)
Supplement: Supplementary file 2 — Additional file 2. Scenario Flowchart data analysis prioritization determinants based on survey data. The appendix describes a flowchart for the decision on the most important determinant per domain of the TICD. [file 12889_2021_11047_MOESM2_ESM.docx]

**Determinants for the implementation of person-centered tools for workers with chronic health conditions: a mixed-method study using the tailored implementation for chronic conditions framework.**

Zipfel, N.^1^, Horreh, B.^1^, Hulshof, C.T.J.^1^, Suman, A.^1 2^, de Boer, A.G.E.M.^1^, van der Burg-Vermeulen, S.J.^1^

1. Amsterdam University Medical Centers, University of Amsterdam, Department of Public and Occupational Health, Coronel Institute of Occupational Health, Amsterdam Public Health research institute, PO Box 22700, 1100 DE Amsterdam, The Netherlands
2. Julius Center for Health Sciences and Primary Care, University Medical Center Utrecht, Utrecht University, Utrecht, The Netherlands

Corresponding author: N. Zipfel, n.zipfel@amsterdamumc.nl

Additional file 2. Scenario Flowchart data analysis prioritization determinants based on survey data. The appendix describes a flowchart for the decision on the most important determinant per domain of the TICD.

Look at the respective TICD domain

Check the frequency count in the first rank

Clear determinant in the first rank (highest frequency count)?

No

Yes

Yes

Select determinant as the most important for the respective domain

No

Frequency count of determinant equal to 1 other determinant?

Check frequency count of respective determinants in the second rank

Frequency count of determinant equal to 2 other determinants?

1 clear determinant in second rank (highest frequency count)?

No

Yes

Yes

Check if determinant with highest frequency count on Rank 2 is identical to one of Rank 1 determinants

No

Yes

Do the same 2 determinants compete on Rank 1 and on Rank 2?

Choose this determinant as top 1

No

Check on Rank 3 which determinant is mentioned the most and is present on Rank 1 and Rank 2

Select both determinants as the most important for the respective domain

Look at the absolute numbers and make a choice thereof (weighted prioritization)
